# Comparison of clinical effects of endoscopic powered osteotome and endoscopic powered drill for UBE-TLIF surgery

**DOI:** 10.1038/s41598-025-08214-9

**Published:** 2025-07-01

**Authors:** Wanlong Xu, Haipeng Si, Yulin Zhao

**Affiliations:** 1 Department of Orthopedics, Qilu Hospital of Shandong University, Cheeloo College of Medicine, Shandong University, Jinan, 250012 Shandong People’s Republic of China; 2 Department of Orthopedics, Qilu Hospital of Shandong University (Qingdao), Cheeloo College of Medicine, Shandong University, Qingdao, 266035 Shandong People’s Republic of China; 3 Key Laboratory of Qingdao in Medicine and Engineering, Department of Orthopedics, Qilu Hospital (Qingdao), Shandong University, Qingdao, 266035 Shandong People’s Republic of China

**Keywords:** Unilateral biportal endoscopic, Lumbar intervertebral fusion, Single-level lumbar spinal canal stenosis, Powered osteotome, Powered drill, Peripheral nervous system, Spine regulation and structure, Musculoskeletal system

## Abstract

This study aims to compare the efficacy of two endoscopic instruments powered osteotome and powered drill in treating single-segment degenerative lumbar spinal stenosis via unilateral biportal endoscopic transforaminal lumbar interbody fusion (UBE-TLIF). We retrospectively analyzed clinical data from 127 patients treated at Qilu Hospital of Shandong University between January 2021 and December 2022. Patients were divided into two groups: the bone-drill (BD) group (71 cases) and the bone-osteotome (BO) group (56 cases). Various surgical indicators were assessed, including operation time, intraoperative blood loss, postoperative drainage volume, length of hospital stay, and complication rates. Clinical efficacy was evaluated using the visual analog scale (VAS) for lower back and limb pain, the Oswestry Disability Index (ODI), modified MacNab criteria, and the Brantigan and Steffee method for interbody fusion assessment. Results showed that the BD group had an average operation time of 151.41 ± 19.03 min, whereas the BO group completed the procedure significantly faster, averaging 128.48 ± 16.92 min. Intraoperative blood loss was comparable between groups (BD: 102.11 ± 34.26 ml; BO: 120.70 ± 32.89 ml). The BO group showed higher postoperative drainage volume (85.47 ± 19.01 ml) than the BD group (71.25 ± 14.55 ml). Hospitalization durations were similar (BD: 8.92 ± 1.22 days; BO: 9.16 ± 1.12 days). Both groups showed significant improvement in VAS and ODI scores at 3 and 12 months post-surgery (*P* < 0.05), with no significant differences between groups (*P* > 0.05). Notably, the BO group exhibited superior intervertebral fusion quality at 3 months compared to the BD group (*P* < 0.05), with no differences observed at 12 months. In conclusion, the UBE-TLIF technique employing a powered osteotome significantly reduces operation time and enhances intervertebral fusion compared to the powered drill method.

## Introduction

In recent years, lumbar spinal stenosis (LSS) has increasingly impaired the health-related quality of life (HR-QoL) of the elderly, surpassing other common comorbidities such as knee and hip osteoarthritis, cardiovascular, cerebrovascular, and respiratory diseases^1^. During lumbar degeneration, spinal stenosis-induced compression is the primary cause of neuropathic pain and dysfunction. Verbiest and Epstein have extensively described congenital and acquired forms of lumbar spinal stenosis^2,3^. Degenerative lumbar spinal stenosis (DLSS), characterized by degenerative changes, is the most prevalent form of LSS. This process is attributed to intervertebral disc dehydration and bulging, along with disc space narrowing due to collapse. These changes increase stress on the facet joints, accelerating cartilage degeneration and osteophyte formation^4,5^. Moreover, degeneration of the intervertebral disc and facet joints can cause central canal or lateral recess stenosis and may result in vertebral displacement, including degenerative spondylolisthesis^6,7^. Such stenotic changes compress nerves, clinically presenting as pain, numbness, fatigue, and gait disturbances in the lower back and legs^8^. Currently, surgery remains the primary treatment for lumbar spinal stenosis. Soliman^9^ improved unilateral biportal endoscopic (UBE) technology by substituting the air medium with fluid irrigation, enhancing surgical field clarity. Consequently, UBE has been widely applied in treating lumbar disc herniation and spinal stenosis^10,11^, and has recently expanded to lumbar fusion with favorable outcomes^12,13^. Unilateral biportal endoscopic transforaminal lumbar interbody fusion causes less trauma than traditional posterior lumbar interbody fusion. By separating observation and operation channels, it significantly enhances surgical flexibility and efficiency^14,15^. Endoscopic bone-drill (BD) is typically used to process the lamina and articular process, allowing efficient removal with minimal blood loss but limited bone preservation^16^. Currently, endoscopic bone-osteotome (BO) is employed to better preserve autologous bone for interbody fusion grafting. This study retrospectively analyzed clinical data from 127 patients with single-segment DLSS who met the inclusion criteria and were hospitalized at Qilu Hospital of Shandong University between January 2021 and December 2022. It compared the efficacy, safety, and follow-up outcomes of two surgical interventions for DLSS, aiming to provide a reference for clinical surgical treatment. The specific findings are presented as follows.

## Materials and methods

### Inclusion criteria

(1) Patients with DLSS presenting typical lumbar segmental symptoms and signs, accompanied by imaging findings consistent with DLSS diagnosis; (2) Lack of improvement after at least 3 months of conservative treatment; (3) Indications met for lumbar fusion surgery. Exclusion criteria: (1) Lumbar spondylolisthesis above grade II or III, vertebral displacement exceeding 3 mm on dynamic lumbar X-rays, or a change in the open angle greater than 13°; (2) Severe lumbar spinal stenosis causing nerve compression and damage; (3) Presence of mental disorders; (4) Severe osteoporosis; (5) Systemic contraindications for surgery; (6) Incomplete clinical or imaging data at 3- and 12-month follow-ups.

### General data

A total of 127 DLSS patients who met the inclusion criteria were enrolled, comprising 67 males and 60 females. All patients underwent UBE-TLIF at our hospital and were divided into the BD group (71 cases) and the BO group (56 cases) based on the endoscopic bone processing technique used. In the BD group, endoscopic bone drill was used to process structures, including the vertebral lamina and articular processes. Due to poor bone preservation, allogeneic bone grafts were implanted into the intervertebral space for fusion. In the BO group, endoscopic bone-osteotome was used to process the vertebral lamina and articular processes, and the autologous bone harvested was implanted into the intervertebral space for grafting and fusion. A written informed consent form is obtained from every patient prior to the surgical procedure. There were no significant differences in baseline characteristics between the two groups (*P* > 0.05), indicating comparability (Table 1).

**Table 1:** Basement data.

Items	Data BD (n = 71)	Data BO (n = 56)	P
Sex (M/F)	39/32	28/28	0.584
Age (years)	63.99 ± 5.07	64.05 ± 5.33	0.930
Levels			
L3/L4	5	2	
L4/L5	41	35	
L5/S1	25	19	
Disease Duration (months)	26.32 ± 17.81	30.29 ± 15.51	0.194
Operation duration (M)	151.41 ± 19.03	128.48 ± 16.92	0.000
blood loss volume(ml)	102.11 ± 34.26	120.70 ± 32.89	0.003
drainage (ml)	71.25 ± 14.55	85.47 ± 19.01	0.011
length of stay(d)	8.92 ± 1.22	9.16 ± 1.12	0.427

### Surgical method

Both groups underwent general anesthesia with the patients placed in the prone position. The responsible spinal segment and pedicle positions were identified and marked under C-arm fluoroscopy. The surgeon stood on the symptomatic side of the patient and made an approximately 1 cm longitudinal incision located 0.5 cm lateral to the spinous process and centered around the intervertebral space. Sequential dilators were used to expand the cannula, through which the observation and working channels were inserted.

A 0° endoscope was introduced through the observation channel. Radiofrequency electrodes were used to achieve hemostasis and clear soft tissues.

**Fig. 1:**
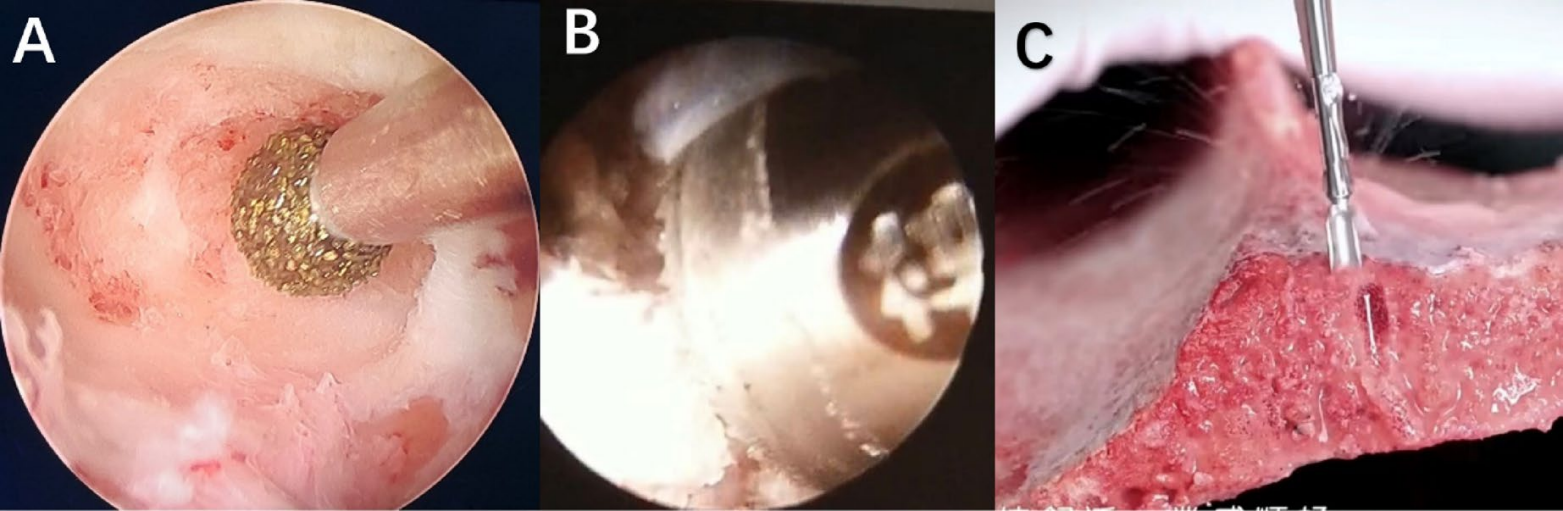
(**A**) Endoscopic bone-drill (BD); (**B**) Endoscopic bone-osteotome (BO); (**C**) Schematic diagram of BO operation on external specimen.

In the BD group, the upper and lower articular processes and part of the vertebral lamina were processed using an endoscopic drill. The ligamentum flavum was resected, and the lateral recess was decompressed to fully expose and protect the nerve roots. Under endoscopic visualization, the annulus fibrosus was incised, the nucleus pulposus was removed, and the endplates were precisely prepared. Subsequently, allogeneic bone grafts and intervertebral fusion devices were implanted (Fig. 1A).

In the BO group, the upper and lower articular processes, along with part of the vertebral lamina, were processed using an endoscopic osteotome. Autologous bone blocks were harvested for subsequent grafting. The ligamentum flavum was resected, and the lateral recess was decompressed to fully expose and protect the nerve roots. The surface of the intervertebral disc was thoroughly hemostatic. Under endoscopic visualization, the annulus fibrosus was incised, the nucleus pulposus was removed, and the endplates were precisely prepared. Autologous bone grafts and an intervertebral fusion device were implanted (Fig. 1B,C; where Fig. 1C illustrates the in vitro specimen procedure schematic).

The nerve roots and dural sac were fully explored. After confirming the size and position of the intervertebral fusion device by fluoroscopy, the endoscope and working channel were withdrawn. Two pedicle screws were percutaneously inserted bilaterally, and connecting rods were applied and fixed. The surgical site was irrigated, drainage was placed, and the wound was closed in layers.

### Perioperative management

Postoperatively, patients received appropriate medications for dehydration, edema reduction, nerve nourishment, and other supportive treatments. Beginning on the first postoperative day, patients were encouraged to mobilize and ambulate under the protection of a rigid lumbar brace. The drainage tube was removed within 48 h after surgery. Patients were advised to primarily rest in bed for the first month postoperatively while performing appropriate back muscle exercises. The lumbar brace was worn for at least 2 months following surgery, and heavy physical labor involving the lumbar region was prohibited for 3 months. Patients were also instructed to strengthen their back muscles through training, correct poor lifestyle habits, and minimize the risk of recurrence. Regular follow-up assessments, including laboratory tests and imaging studies, were performed to monitor recovery.

### Evaluation indicators

The operation time, intraoperative blood loss, postoperative drainage volume, length of hospital stay, and complication rates were statistically analyzed and compared between the two groups. Baseline preoperative data were also compared to ensure group comparability. Visual Analog Scale (VAS) scores for low back pain were collected preoperatively, as well as at 3 months and 12 months postoperatively, to evaluate treatment efficacy. Clinical outcomes at the final follow-up were assessed using the modified MacNab criteria to determine excellent and good rates. Additionally, spinal fusion status was evaluated according to the Brantigan and Steffee fusion grading system^17,18^ (Table 2). Postoperative fusion evaluation in this study was conducted by our center’s team of physicians. Assessors reviewed imaging data at 3 and 12 months postoperatively, with specific surgical methods anonymized to prevent bias. Two physicians independently assessed the fusion status, and any discrepancies were resolved through discussion to reach consensus, thereby minimizing subjective bias.

**Table 2:** Brantigan and Steffee spine fusion classification.

Grade	Characteristics
A	Obvious structural collapse due to pseudoarthrosis, loss of intervertebral height, vertebral slippage, broken screws, cage displacement, and bone graft absorption
B	Significant bone resorption, possibly due to pseudoarthrosis, with large radiolucencies or visible fissures in the fusion area (2 mm from all implant edges)
C	In an indeterminate nonunion, the density of the graft in the fusion area is comparable to that immediately after surgery. Small radiolucencies or cracks are visible in at least half of the fusion area
D	The entire bone graft area can be seen with bone bridges that may be fused and have a density equivalent to that immediately after surgery. There is no translucent zone between vertebral bodies. The bone density in the fusion area is greater than that immediately after surgery
E	Even if a sclerotic line between the implant and the vertebral body indicates fusion, ideally there should be no clear demarcation between the implant and the vertebral body. Other signs of fusion include bone bridging within the fusion zone, resorption of anterior bone spurs, and fusion of facet joints

### Statistical methods

IBM SPSS Statistics ver. 22.0 (IBM Co., Armonk, NY, USA) was used for statistical analysis. For continuous variables, the mean values are presented with standard deviations, and the differences between the 2 groups were analyzed using the Student t-test. A chi-square or Fisher exact test was used to assess associations between dichotomous categorical variables. The Kruskal–Wallis H test was used for multiple categorical variables. A *p*-value of < 0.05 was considered statistically significant. The intraclass correlation coefficient (ICC) was used to evaluate the intrarepeatability of different observers (interobserver reliability).

## Results

A total of 127 patients with degenerative lumbar spinal stenosis (DLSS) were included, comprising 67 males and 60 females. All patients underwent unilateral biportal endoscopic transforaminal lumbar interbody fusion (UBE-TLIF) at our hospital. The surgical segments involved were L3/L4 in 47 patients, L4/L5 in 76 patients, and L5/S1 in 4 patients. Based on the microscopic bone treatment method, patients were divided into the bone drilling (BD) group (71 cases) and bone osteotome (BO) group (56 cases).

The mean age was 63.99 ± 5.07 years in the BD group and 64.05 ± 5.33 years in the BO group, with no significant difference. The duration of medical history was 26.32 ± 17.81 months in the BD group and 30.29 ± 15.51 months in the BO group, also showing no statistically significant difference. Perioperative data comparison revealed the following:

- Operation time: 151.41 ± 19.03 min in the BD group and 128.48 ± 16.92 min in the BO group (*P* < 0.05, shorter in the BO group)
- Intraoperative blood loss: 102.11 ± 34.26 ml in BD group versus 120.70 ± 32.89 ml in BO group (*P* < 0.05, higher in BO group)
- Postoperative drainage volume: 71.25 ± 14.55 ml in the BD group compared to 85.47 ± 19.01 ml in the BO group (*P* < 0.05, higher in the BO group)
- Length of hospital stay: 8.92 ± 1.22 days in the BD group and 9.16 ± 1.12 days in the BO group (no significant difference)

In summary, compared with the BD group, the BO group had a significantly shorter operation time, but greater intraoperative blood loss and postoperative drainage volume. Hospital stay was similar between groups (Table 1).

127 patients were followed up for 3 months and 12 months, respectively. The VAS scores and ODI scores of the two groups were compared with the VAS scores and ODI scores of low back and leg pain before surgery, and the scores at each time point after surgery were significantly reduced (*P* < 0.05). At the same time, the scores between the groups after surgery were compared, and the results showed that there was no statistically significant difference in the VAS scores of low back pain (*P* > 0.05), and there was a statistically significant difference in the VAS scores and ODI index of leg pain (*P* < 0.05). The specific data are shown in Table 3. One case of postoperative lower limb numbness occurred in the BD group after surgery, which was improved after dehydration and nerve nutrition drugs. However, there was no statistically significant difference in the incidence of postoperative complications between the two groups (χ2 < 0.001, *P* = 1.000); the MacNab efficacy evaluation at the last follow-up was 94.36% (67/71) in the BD group and 94.64% (53/56) in the BO group, with no statistically significant difference (*P* > 0.05).

**Table 3:** Brantigan and Steffee spinal fusion grades at 3 and 12 months after surgery.

Follow-up	Group	BD(n = 71)					BO(n = 56)					*H*	*P*
	Grade	A	B	C	D	E	A	B	C	D	E		
3 months	L3/4(n = 6/2)	0	2	4	0	0	0	0	1	1	0	5.549	0.018
	L4/5(n = 41/35)	0	6	25	9	0	0	0	20	15	0		
	L5/S1(n = 25/19)	0	2	17	6	0	0	2	13	4	0		
	total	0	10	46	15	0	0	2	34	20	0		
12 months	L3/4(n = 6/2)	0	0	1	3	1	0	0	1	1	0	3.285	0.070
	L4/5(n = 41/35)	0	0	4	25	12	0	0	1	11	21		
	L5/S1(n = 25/19)	0	0	2	14	9	0	0	1	8	10		
	total	0	0	7	42	22	0	0	3	19	21		

Kruskal–Wallis H test: Comparing the fusion levels between groups. H: Test statistic, P: Significance probability value, with P ≤ 0.05 indicating that the difference is statistically significant.

Comparison of fusion rates and excellent/good clinical outcomes between the two groups at 12 months postoperatively showed that all surgical segments achieved fusion of grade 4 or above. At the 3-month follow-up, fusion grading in the BD group was: grade A (0 cases), B (10 cases), C (45 cases), D (15 cases), and E (0 cases). In the BO group, the distribution was: grade A (2 cases), B (34 cases), C (20 cases), D (4 cases), and E (0 cases). At the 12-month follow-up, the BD group had fusion grades: A (0 cases), B (0 cases), C (7 cases), D (42 cases), and E (22 cases), while the BO group had: A (0 cases), B (0 cases), C (3 cases), D (19 cases), and E (21 cases) (Table 4).

**Table 4:** Comparison of scores among groups after surgery.

score	Group	Preoperative	3 months postoperative	*P1*	12 months postoperative	*P2*
VAS (Lumbar)	BD	4.63 ± 0.78	1.99 ± 0.62	0.000	1.24 ± 0.55	0.000
	BO	5.09 ± 0.79	2.09 ± 0.69	0.000	1.20 ± 0.52	0.000
VAS (Leg)	BD	5.46 ± 0.89	1.92 ± 0.91	0.000	1.11 ± 0.77	0.000
	BO	5.46 ± 0.82	2.05 ± 0.62	0.000	0.69 ± 0.79	0.000
ODI	BD	34.08 ± 3.37	17.37 ± 2.59	0.000	12.21 ± 1.58	0.000
	BO	33.34 ± 3.13	16.86 ± 2.05	0.000	11.86 ± 1.63	0.000

VAS, Visual Analog Scale; ODI, Lumbar Oswestry Disability Index; *P*1, the *P*-value between 3-month postoperative and pre-operative; *P*2, the *P*-value between 12-month postoperative and pre-operative.

The Kruskal–Wallis H test was used to compare the fusion grades between groups. At 3 months, the test statistic was 5.549 with a *P* value of 0.018, indicating a statistically significant difference between the groups (*P* < 0.05), with the BO group showing superior fusion quality. At 12 months, the test statistic was 3.285 with a *P* value of 0.070, showing no significant difference in fusion quality between groups. These results suggest that although the BO group demonstrated significantly better fusion quality at 3 months, fusion outcomes were comparable between the groups at 12 months.

## Discussion

With the continuous trend of population aging, the prevalence of degenerative lumbar spinal stenosis (DLSS) has further increased, significantly impacting patients’ daily lives and work, and imposing a growing social and economic burden. Currently, minimally invasive surgery represents a mainstream and evidence-based approach for treating lumbar spinal stenosis. In recent years, unilateral biportal endoscopic transforaminal lumbar interbody fusion (UBE-TLIF) has gained widespread attention among spinal surgeons. Owing to its relatively flat learning curve, Kim et al.^19^ retrospectively analyzed 57 cases of UBE-TLIF and found that operative time and postoperative outcomes stabilized after the 34th case, with average operation time significantly reduced from 193.4 ± 28.3 min before the learning curve plateau to 139.7 ± 11.6 min afterward. HEO et al.^14^ noted that the endoscopic view provides a more comprehensive surgical field, and the instruments used in UBE-TLIF are similar to those in traditional open lumbar fusion but allow for more flexible manipulation. These advantages make UBE-TLIF highly effective for treating lumbar spinal stenosis. Moreover, in patients with bilateral spinal stenosis, UBE-TLIF can achieve effective bilateral decompression. After completing ipsilateral decompression, the endoscope is angled medially, and a plasma radiofrequency blade is used to expose the root of the superior spinous process. Subsequently, a power drill is employed to remove bone tissue at the base of the spinous process to expose the interspinous ligament and bilateral ligamentum flavum for contralateral decompression.

According to the perioperative data, surgeries in both groups were completed successfully without major complications. Notably, the operation time in the BO group was longer than in the bone drilling (BD) group. This difference is partly due to surgeon familiarity with the BD technique under the microscope. The BO allows removal of bone tissue in a single piece, and with further optimization of the technique, operative time is expected to decrease. Previous studies have reported that UBE-TLIF is associated with reduced intraoperative bleeding, with average blood loss around 170 ml^20^. However, in our study, the BO group had significantly greater intraoperative blood loss and postoperative drainage volume compared to the BD group. This may be attributed to the BD technique generating high local heat, which reduces bleeding, whereas the BO technique results in a fresh bone surface that is more prone to bleeding^21^. Regarding postoperative hospital stay, no significant difference was observed between the two groups, indicating that both techniques maintain the benefits of minimally invasive surgery, including rapid recovery^22^.

Comparison of clinical efficacy indicators showed that the Visual Analog Scale (VAS) scores and Oswestry Disability Index (ODI) for low back pain at 3 months and 12 months postoperatively were significantly lower than preoperative scores in both groups. There was no statistically significant difference between the two groups in VAS scores for low back pain at any time point before or after surgery. From a safety perspective, one case of postoperative lower limb numbness occurred in the BD group, which resolved following dehydration and neurotrophic therapy. This complication was likely related to excessive nerve root traction during surgery, causing transient nerve root edema. Overall, there was no statistically significant difference in complication rates between the two groups (*P* > 0.05), indicating that both surgical procedures have a reliable safety profile^22^. The grouping in this study was performed retrospectively based on the different surgical methods undertaken by patients; in fact, both surgical methods were used concurrently at that time. The choice of which method to use primarily depended on the surgeon’s familiarity with the technique and the specific circumstances of the patient. The selection process was relatively random, and there was no fixed rule designating a certain patient type for a specific surgical method. Therefore, while the grouping was based on surgical techniques, it was not a deliberate allocation based on a specific type of patient. Of course, the fine operation under microscopy is closely related to the surgeon’s proficiency and surgical experience. The surgeons for both groups in this study were all from the same center and of the same level, eliminating deviations in safety due to differences in the surgeon’s skill level.

Accurate evaluation of postoperative lumbar interbody fusion is critical for assessing prognosis^23,24^. Although endoscopic fusion techniques have demonstrated satisfactory clinical outcomes, numerous studies report that the fusion rates of unilateral biportal endoscopic (UBE) fusion are comparable to those achieved with open transforaminal lumbar interbody fusion (TLIF)^25,26^. In this study, we compared the interbody fusion rates and excellent clinical outcomes of two patient groups undergoing UBE-TLIF at 3 and 12 months postoperatively. The interbody fusion rate exceeded 85% at 12 months in both groups, indicating that both approaches achieve satisfactory fusion and clinical efficacy, with reliable outcomes overall. Notably, the BO group demonstrated superior fusion grades compared to the BD group at both 3 and 12 months, suggesting faster healing and a more robust fusion effect with the BO technique.

In this study, the BO group utilized an endoscopic powered osteotome featuring a high-speed reciprocating blade motion for bone cutting. This instrument fundamentally differs from traditional powered drills and conventional ultrasonic bone scalpels. Ultrasonic bone scalpels function by generating high-frequency longitudinal vibrations at the tip, enabling simultaneous bone cutting and hemostasis. In contrast, the endoscopic powered osteotome employs high-frequency, low-amplitude reciprocating mechanical motions at speeds reaching up to 60,000 rpm, allowing rapid bone cutting with a blade thinner than that of ultrasonic scalpels. This design effectively preserves more bone tissue, which can be retained intact for intervertebral bone grafting. Previously, Kerrison punches were commonly used to treat the vertebral lamina while preserving autologous bone; however, this technique is relatively inefficient^12,22^. Regarding overall operative cost, the BO group was able to reduce the volume of allogeneic bone graft implantation by approximately 5–10 cm^3^ compared to the BD group, presuming equivalent costs for other aspects of the procedure.

This study was a single-center, retrospective clinical investigation with a limited sample size, short follow-up duration, and focused exclusively on single-segment degenerative lumbar spinal stenosis. These limitations highlight the need for future research involving larger cohorts, longer follow-up periods, and evaluation of multi-level degenerative cases to enhance the generalizability of the findings.

In summary, both procedures are minimally invasive endoscopic fusion techniques that demonstrate satisfactory clinical efficacy in relieving pain, improving lumbar function, and achieving high rates of excellent or good outcomes and intervertebral fusion at the final follow-up in the treatment of degenerative spinal stenosis. However, the BO technique offers advantages including shorter operative time and a higher postoperative lumbar fusion rate, aligning well with current principles of precision and minimally invasive surgery. Additionally, compared to the BD group, the BO technique does not impose greater demands on the surgeon’s learning curve or surgical precision, making it more accessible and easier to implement in clinical practice.

## Data Availability

The data that support the findings of this study are available from the corresponding author, Zhao upon reasonable request.
